# Innovative Strategy for Developing PEDOT Composite Scaffold for Reversible Oxygen Reduction Reaction

**DOI:** 10.1021/acs.jpclett.4c00482

**Published:** 2024-04-26

**Authors:** Rafael Del Olmo, Antonio Dominguez-Alfaro, Jorge L. Olmedo-Martínez, Oihane Sanz, Cristina Pozo-Gonzalo, Maria Forsyth, Nerea Casado

**Affiliations:** † POLYMAT, University of the Basque Country UPV/EHU, Joxe Mari Korta Center, Tolosa 72, 20018 Donostia-San Sebastián, Spain; ‡ Department of Applied Chemistry, University of the Basque Country UPV/EHU, 20018 Donostia-San Sebastián, Spain; § Institute for Frontier Materials (IFM), Deakin University, Burwood, Victoria 3125, Australia; ∥ Ikerbasque, Basque Foundation for Science, E-48011 Bilbao, Spain

## Abstract

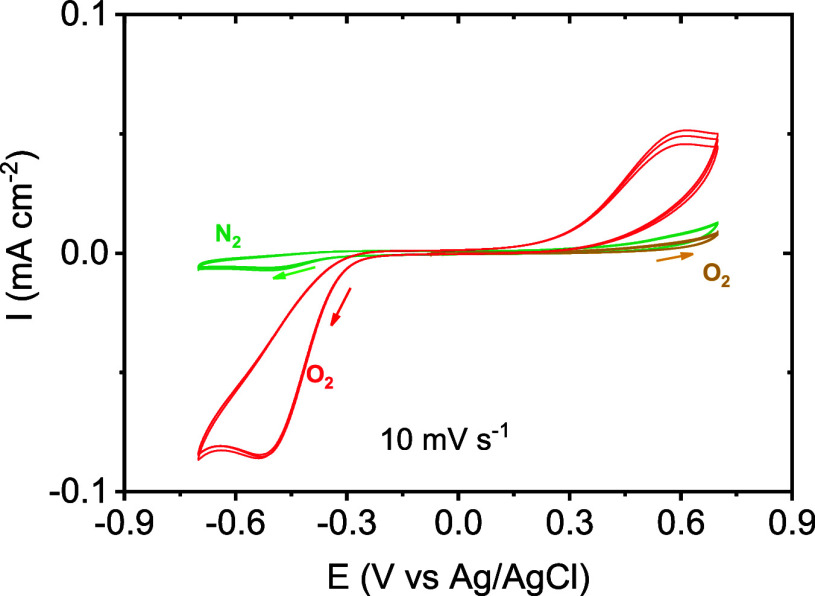

Metal–air batteries are an emerging technology with great potential to satisfy the demand for energy in high-consumption applications. However, this technology is still in an early stage, facing significant challenges such as a low cycle life that currently limits its practical use. Poly(3,4-ethylenedioxythiophene) (PEDOT) conducting polymer has already demonstrated its efficiency as catalyst for oxygen reduction reaction (ORR) discharge as an alternative to traditional expensive and nonsustainable metal catalysts. Apart from that, in most electrochemical processes, three phenomena are needed: redox activity and electronic and ionic conduction. Material morphology is important to maximize the contact area and optimize the 3 mechanisms to obtain high-performance devices. In this work, porous scaffolds of PEDOT–organic ionic plastic crystal (OIPC) are prepared through vapor phase polymerization to be used as porous self-standing cathodes. The scaffolds, based on abundant elements, showed good thermal stability (200 °C), with potential ORR reversible electrocatalytic activity: 60% of Coulombic efficiency in aqueous medium after 200 cycles.

Metal–oxygen batteries are a promising alternative to lithium-ion batteries (LIB) for automotive applications due to their high theoretical energy density (3500 Wh kg^–1^ for lithium–air batteries, which is superior to that of gasoline 1700 Wh kg^–1^).^1−5^ Nonetheless, many challenges need to be solved before their commercialization becomes a reality. Air cathodes are generally considered the main bottleneck for the performance of metal–O_2_ systems. They need to catalyze not only the discharge reaction, i.e., oxygen reduction reaction (ORR), but also the reversible charge process, i.e., oxygen evolution reaction (OER). In the ORR step, peroxide byproducts are typically generated, which need to be established carefully to avoid dissolution into the electrolyte to allow the reversibility of the subsequent OER process. An optimum air cathode should provide high oxygen diffusion and enough active sites to favor the ORR, accommodating at the same time a large amount of discharge products generated during battery operation.^6,7^ In addition, the electrode material must offer thermal, chemical, and electrochemical stability and high electronic conductivity to build safe devices with minimum energy losses. Apart from the selection of good ORR/OER catalysts for high coulombic efficiencies and long-term stability, other parameters like oxygen diffusion through the surface structure, interfacial reactions (electrode–electrolyte), and electrolyte stability are also important.^8,9^

Carbon materials with a 3D porous structure are widely studied for air cathodes, presenting a cost-effective alternative to replacement of high-cost and scarce noble metals.^10−12^ Among them, we can find 3D-ordered porous carbon,^13−15^ carbon nanotubes,^16−19^ graphene,^20−22^ and conducting polymer derivatives.^23−27^ Despite having abundant sources and being lightweight, they present low catalytic activity toward the OER in aqueous-based electrolytes. Past works have balanced this drawback by including dopants, like nitrogen,^28−30^ boron,^31,32^ phosphorus,^33^ or metals,^34,35^ in the structure. Winther-Jensen et al. proposed poly(3,4-ethylenedioxythiophene) (PEDOT) as an effective ORR catalyst under alkaline conditions.^26^ PEDOT was prepared by vapor phase polymerization (VPP) onto a porous surface showing a continuous operation for 1500 h with no degradation, comparable with Pt-catalyzed electrodes, and no signs of poisoning in the presence of CO. VPP has been widely used to control the coating formation of conducting polymers for high-performance applications in a cheap and versatile way. The method consists basically of the evaporation of a monomer solution and subsequent polymerization onto a substrate that already contains the oxidative initiator as described in the literature.^36^ Subsequently, Kerr et al. investigated in more detail the ORR activity of PEDOT with different deposition techniques (VPP and electrodeposition), observing that the ORR with VPP-PEDOT undergoes a transition from a 2-electron pathway to a 4-electron pathway at −0.45 V (vs Ag/AgCl) while the ORR with electrodeposited-PEDOT proceeds only by 2-electron pathway.^27^ There are several studies investigating OER activity of PEDOT with additives such as CoMn_2_O_4_ or CoNi_2_S_4_, but they present the same drawbacks mentioned before, regarding the use of scarce metals.^37,38^ Moreover, all of the above-mentioned works rely on PEDOT-deposited thin electrodes, without exploiting the potential of large effective electrode surface area through porous 3D architectures. It is well-known that high surface area in air cathodes offers more active sites to adsorb oxygen on the surface for the ORR.^39^

Recently, an innovative way of manufacturing self-standing scaffolds made of conducting polymers (CP) such as PEDOT or polypyrrole with a controllable porosity at the nanoscale through VPP was reported.^40,41^ In these works, CPs are polymerized surrounded by sucrose, an oxidant, and carbon nanotubes (CNT) to strengthen the resultant structure. Subsequently, the material is washed to remove the oxidant and sucrose and produce a scaffold. On the other hand, organic ionic plastic crystals (OIPCs), which are considered the solid-state version of ionic liquids, have attracted attention as electrolytes due to their high ionic conductivity while remaining in their solid state.^42,43^ Ionic liquids containing quaternary ammonium- and phosphonium-based cations have emerged as a promising materials to stabilize superoxide anion forming complexes and consequently improve the OER.^8,44^

Previous works reported the use of OIPCs as functional binders for anodes and cathodes, where good mechanical properties are required for satisfactory performance, being even successfully combined with PEDOT to produce mixed ionic and electronic conductors (MIECs).^45−47^ These latter materials are able to provide both electronic and ionic pathways in an electrode structure, in addition to catalytic behavior previously discussed for PEDOT. Moreover, pyrrolidinium-based electrolytes like *N*-butyl-*N*-methylpyrrolidinium bis(trifluoromethanesulfonyl)imide [C_4_mpyr][TFSI] have enabled very stable operation for ORR/OER.^48^ Herein we propose porous scaffolds based on using an OIPC (*N*-ethyl-*N*-methylpyrrolidinium bis(trifluoromethanesulfonyl)imide, [C_2_mpyr][TFSI]) as a dopant for PEDOT that is produced by vapor phase polymerization, in a similar way as described by Dominguez et al.^41^ [C_2_mpyr][TFSI] is hypothesized to not only act as the scaffold for the electrode structure but also provide ionic conductivity very desirable in air cathodes. Additionally, those scaffolds lead to more free space to accommodate discharge products, which could be very promising for metal–air batteries using nonaqueous media. Different ratios of OIPCs to the scaffolding components, sucrose and FeCl_3_ initiator, during the VPP process were prepared and correlated to the porosity and electrochemical properties. Thermogravimetric analysis (TGA) was used to estimate the PEDOT/OIPC composition, and the morphology of the scaffolds was investigated by N_2_ physisorption and scanning electron microscopy (SEM). Finally, the more porous scaffolds were studied electrochemically for ORR/OER application.

The main goal of this study is to obtain a material with a high porosity and electrochemical response while maintaining a self-standing 3D PEDOT/OIPC scaffold. Figure 1 shows an overview of the synthetic procedure to produce PEDOT/OIPC scaffolds, which was described in detail in the Experimental Section. The polymerization consists of a chemical oxidative polymerization in the vapor phase (VPP) in which PEDOT was polymerized within the interstices of a sucrose template. According to VPP methods, the temperature was increased (140 °C) forming the EDOT vapor. The polymerization took place within the interstices of the template, where the EDOT and the oxidant came into contact. In the VPP system, iron(III) was used as oxidant, sucrose as porous template, and OIPC as the element that confers plasticity to the 3D structure. After the polymerization, the material was cleaned of sucrose and oxidant impurities as indicated in the Experimental Section with water and isopropanol to obtain the final porous PEDOT/OIPC scaffolds. In order to evaluate the effect of OIPC on the scaffold properties, different amounts of sieved OIPC (100–250 μm) were employed: 20, 40, and 80 mg (here named SC-20, SC-40, and SC-80), as represented in Figure 1.

**Figure 1:**
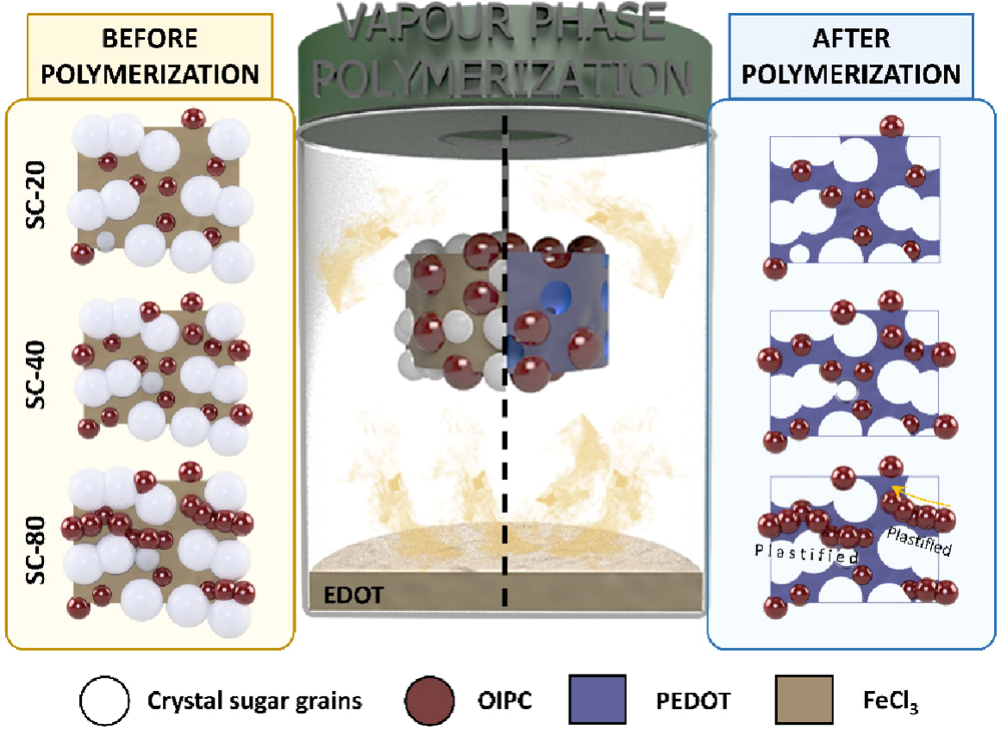
General scheme of PEDOT/OIPC scaffolds manufactured through VPP.

The thermal properties of the scaffolds were investigated with TGA and DSC. TGA was employed to evaluate the ratio of PEDOT/OIPC for the three prepared scaffolds based on the different amount of OIPC. Two distinctive, well-separated degradation curves were observed (Figure 2a and first-derivative curves in Figure S1); the first centered at 400 °C, related to PEDOT polymer loss, and a second centered close to 600 °C, corresponding to [C_2_mpyr][TFSI] OIPC.^41,49^ Interestingly, the scaffolds present a small first step of degradation around 200 °C which is related to the decomposition of an interphase PEDOT–OIPC component also observed in a similar work based on the solid mixing of PEDOT-Cl + [C_2_mpyr][FSI] OIPC.^47^ In this first step of decomposition, the scaffolds show a different peak shape in terms of intensity, probably related to different degrees of PEDOT–OIPC interaction during the EDOT polymerization as a consequence of the different amount of OIPC used in the template.

**Figure 2:**
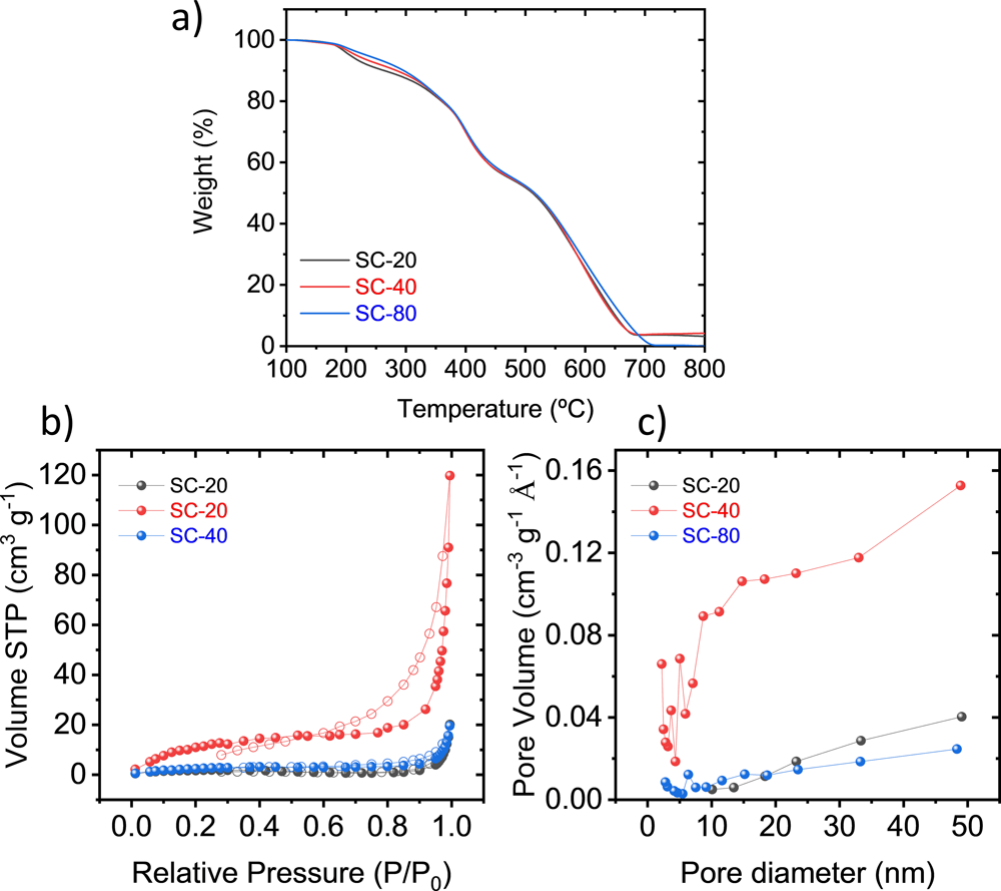
(a) TGA under air of SC-20, SC-40, and SC-80 as indicated in the figure legend at 10 °C min. (b) N_2_ physisorption isotherms of SC-20, SC-40, and SC-80. The full and empty symbols correspond to adsorption and desorption branches, respectively. (c) Pore size distribution obtained from N_2_ physisorption.

The amount of PEDOT in the scaffold was estimated in terms of percentage of weight loss at 470 °C, which is before the degradation of the OIPC. The PEDOT content in all of the scaffolds was around 45 wt %, suggesting that the OIPC content in the template determines the structure porosity of the scaffold, but not the total PEDOT/OIPC content, as the excess of the OIPC was removed during the cleaning step.

Differential scanning calorimetry (DSC) was performed to analyze the changes in the OIPC phases (Figure S2). Neat [C_2_mpyr][TFSI] shows several solid–solid phase transition temperatures with a melting point at 90 °C, but the scaffolds presented no thermal transitions, suggesting an amorphous state of the OIPC in good agreement with reported PEDOT-Cl/[C_2_mpyr][FSI] mixtures at this PEDOT composition.^47^ This fact is typically beneficial for the ion transport within the material since the OIPC present higher ionic conductivities in their amorphous state.

One of the key features of an air electrode is to present a high surface area to have a larger number of active sites for the reduction of O_2_. Nitrogen physisorption was employed to evaluate the porosity of the three scaffolds. As shown in Figure 2b,c, the scaffolds exhibit type II isotherms without an increase at *P*/*P*_0_ < 0.01, implying the absence of significant microporous structure. The N_2_ uptake increased at medium relative pressures, corresponding to the filling of mesopores of N_2_.^50^ Moreover, there is a big rise at *P*/*P*_0_ > 0.8 revealing a higher contribution of large mesopores/macropores rather than micropores and narrow mesopores. The results show a clear difference between the different materials, obtaining for SC-40 the highest specific surface area (SSA) and estimated porosity (45.2 m^2^ g^–1^, 22.2%), followed by SC-80 (10.2 m^2^ g^–1^, 3.6%) and SC-20 (5.5 m^2^ g^–1^, 3.7%) considering an estimated apparent density of 1.2 g mL^–1^. Comparable values in the range of 20–25 m^2^ g^–1^ were obtained for carbon nanofiber air cathodes based on poly(diallyldimethylammonium) with similar chemistry.^39^ Interestingly, the equivalent pore diameter decreases with the amount of OIPC incorporated in the formulation (22.6, 16.4, and 11.9 nm for SC-20, SC-40, and SC-80, respectively), probably due to its plasticizing behavior.^46^

The morphology of the PEDOT/OIPC scaffolds was further analyzed by scanning electron microscopy (SEM). As can be seen from Figure 3, SC-20 presents a heterogeneous surface with different sizes of pores and a rigid appearance. In contrast, SC-80 appears more amorphous with less well-defined porous structure. Furthermore, in handling the material, it was significantly less brittle. This could be due to the agglomeration of the OIPC on the surface of the scaffold. Finally, SC-40 is in between both scenarios, exhibiting a homogeneous porous structure in accordance with Brunauer–Emmett–Teller (BET) experiments. The pore size distribution (see Figure S3) shows that the pores obtained in SC-20 vary from 1 to 20 μm while in SC-40 and SC-80 the pores are smaller mainly in the range between 1 and 5 μm.

**Figure 3:**
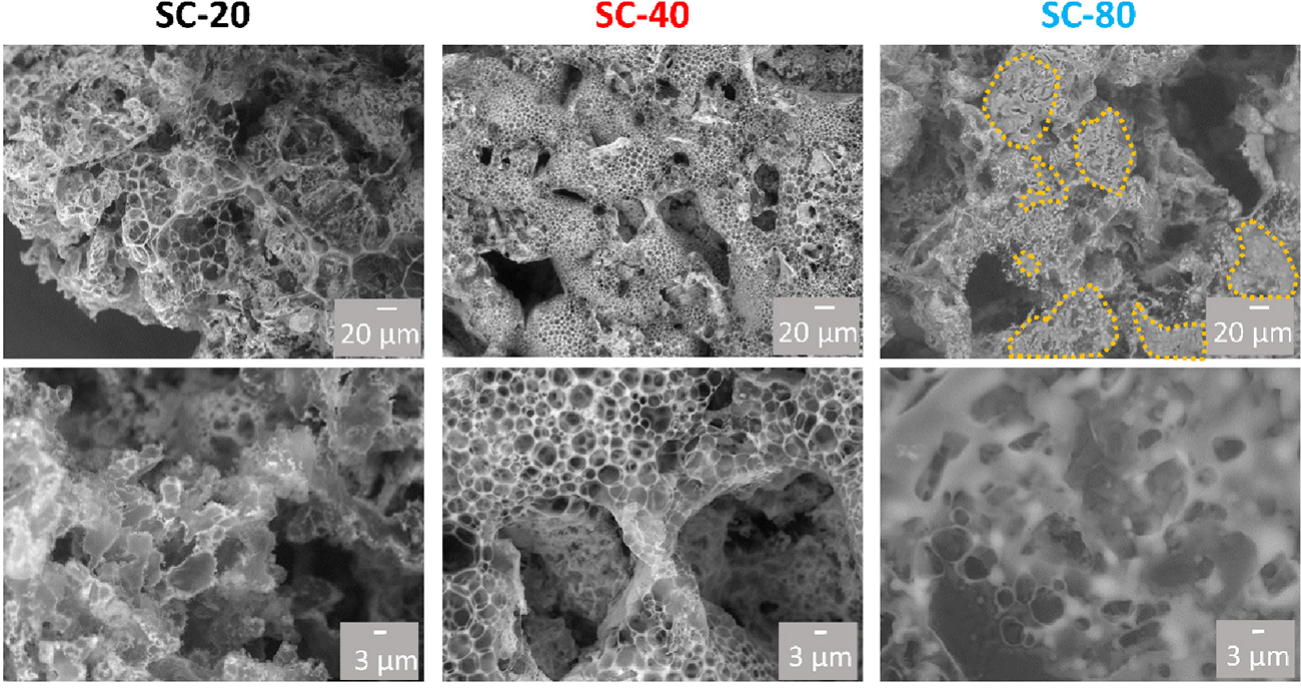
SEM images of SC-20, SC-40, and SC-80 scaffolds at different magnifications. Orange dashed lines highlight the plasticized areas for SC-80.

Given the fragile nature of SC-20, only the electrocatalytic activities of SC-40 and SC-80 scaffolds were evaluated in an oxygen saturated basic medium (0.1 M KOH) from −0.7 to 0.7 V vs Ag/AgCl. As observed in Figure 4, SC-40 and SC-80 were compared against different materials at 10 mV s^–1^. Platinum (Pt) and glassy carbon (GC) exhibited one well-defined peak, in each case corresponding to the oxygen reduction at negative voltages: −0.05 and −0.30 V versus Ag/AgCl, respectively. In contrast, SC-40 presents a broader peak at −0.52 V similar to commercial PEDOT:PSS (−0.47 V). This peak has been also observed as a shoulder in previous works based on PEDOT synthesized by VPP at ∼−0.45 V.^27^ SC-80 does not present this well-defined peak but exhibits a more positive onset potential (−0.13 V) than SC-40 (−0.32 V) indicating a greater ability to reduce oxygen. Considering the very similar composition between SC-40 and SC-80, the main difference is attributed to the different SSAs and pore sizes.

**Figure 4:**
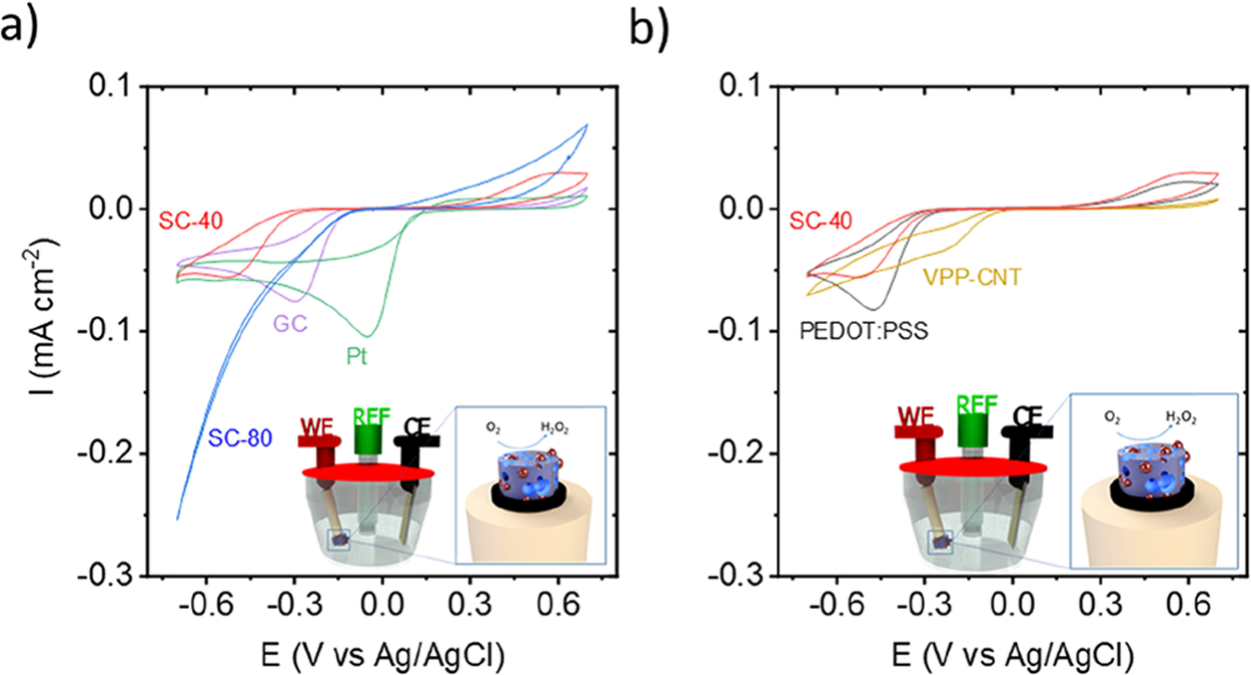
Cyclic voltammograms of (a) SC-40 scaffold (15.4 mg), glassy carbon (GC), and platinum (Pt) electrodes of 0.126 cm^–2^ and (b) SC-40, PEDOT:PSS (15 μL) and VPP-CNT. The experiments were performed in an oxygen-saturated basic medium (0.1 M KOH) at 10 mV s^–1^ (geometrical area of electrodes 0.126 cm^–2^).

As observed from Table 1, considering the capacity obtained from the integral of current vs time curves, the SC-80 scaffold obtained the highest ORR signal (4.43 ± 0.09 μAh cm^–2^) followed by Pt, GC, PEDOT:PSS, and finally SC-40 (3.89 ± 0.28, 2.03 ± 0.08, 1.57 ± 0.04, and 1.17 ± 0.05 μAh cm^–2^, respectively). Moreover, it is worth considering the magnitude of the peak at positive potentials related to the reverse process of the ORR, e.g., oxygen evolution reaction (OER). In this way, the SC-40 scaffold enabled the highest Coulombic efficiency (51.55 ± 2.04%) among the samples investigated in this work, followed by SC-80 and PEDOT:PSS (25.17 ± 0.42% and 24.8 ± 2.45%) while Pt and GC remained significantly lower (5.61 ± 0.30% and 8.11 ± 0.30%). When comparing SC-40 and planar PEDOT:PSS, we can observe similar current densities, except that the Coulombic efficiency is doubled. This improvement could be due to the porous structured scaffold or the presence of OIPC as discussed further below. For all samples, the shape of the electrochemical response was maintained over 6 cycles as can be seen from Figure S4, and the electrochemical response was attributed entirely to oxygen species since the response under a N_2_ atmosphere did not show any redox processes.

**Table 1:** Electrochemical Behavior of Glassy Carbon (GC), Platinum (Pt), SC-40, SC-80, PEDOT:PSS, and VPP-CNT^a^

material	ORR peak position (V vs Ag/AgCl)	ORR onset position (V vs Ag/AgCl)	Cathodic current density (μAh cm^–2^)	Anodic current density (μAh cm^–2^)	Coulombic efficiency (%)
GC	–0.30	–0.15	2.03 ± 0.08	0.16 ± 0.01	8.11 ± 0.30
Pt	–0.05	–0.07	3.89 ± 0.28	0.22 ± 0.01	5.61 ± 0.30
SC-40	–0.52	–0.32	1.17 ± 0.05	0.59 ± 0.02	51.55 ± 2.04
SC-80	not defined	–0.13	4.43 ± 0.09	1.12 ± 0.04	25.17 ± 0.42
PEDOT:PSS	–0.47	–0.31	1.57 ± 0.04	0.39 ± 0.03	24.80 ± 2.45
VPP-CNT	not defined	–0.07	2.03 ± 0.05	0.08 ± 0.01	3.74 ± 0.32

a Error bars are calculated considering 6 cycles.

Given the outstanding efficiency of SC-40, scaffolds based on PEDOT and CNT (VPP-CNT) were synthesized following the previously reported work to assess if it was related to the porous structure for comparison.^41^ In this case, VPP-CNT (Figure 4b) presented a shoulder in the ORR current around −0.15 V but a very low OER response (see Figure S6 for gravimetric current), leading to a Coulombic efficiency of 4.4%. Even if SSA and pore size distribution can play a determinant role as observed between SC-40 and SC-80, the presence of OIPC is certainly helping with the OER according to PEDOT-CNT scaffold results.

To study SC-40 in more detail, additional experiments were undertaken. Figure 5a shows the electrochemical response of a fresh scaffold, where it can be observed that no signal is present in a nitrogen-saturated solution or when cycling only at positive voltages; this indicates that all the observed redox processes are related to the oxygen activity. After cycling SC-40 at negative potentials, under oxygen, the anodic peak related to the oxidation of peroxide species appears, showing the correlation between the reduction and oxidation process. To elucidate the role of peroxides species in the system, the scaffold was subsequently cycled in an oxygen saturated basic medium at 20 mV s^–1^, and subsequently 1 mL of H_2_O_2_ was introduced. As observed in Figure 5b, the electrochemical response was increased until the second or third cycle. In the presence of H_2_O_2_, SC-40 can oxidize the peroxides to oxygen in the same range of positive voltages. Subsequently, the extra oxygen species generated can be reduced again. Besides, this experiment shows that the SC-40 scaffold took several cycles for the internal porous structure to become saturated with oxygen.

**Figure 5:**
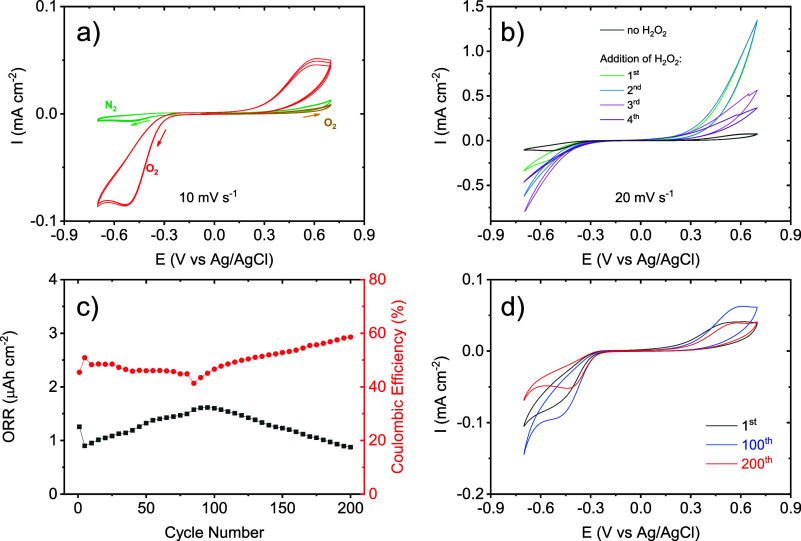
(a) Cyclic voltammograms of SC-40 scaffold (8.5 mg) using 0.1 M KOH with nitrogen- and oxygen-saturated solutions at 10 mV s^–1^. (b) Cyclic voltammograms of SC-40 in oxygen-saturated 0.1 M KOH solution (black line) and subsequently addition of H_2_O_2_ at 20 mV s^–1^. Potentiostatic long-term cycling of SC-40 (8.5 mg) at 10 mV s^–1^ in an oxygen-saturated 0.1 M KOH aqueous electrolyte. (c) Capacity of ORR and Coulombic efficiency obtained. (d) Cyclic voltammetry shape at different cycles.

The stability of reversible ORR upon cycling of the SC-40 scaffold was measured with a fresh scaffold by cyclic voltammetry (Figure 5c,d). The scaffold exhibited a stable cycling of 200 cycles showing an increasing capacity in both anodic and cathodic peaks during the first 100 cycles which could be explained considering the saturation of the entire scaffold by the products being trapped (but electrochemically accessible). Despite the slight drop in ORR capacity observed in the next cycles (100–200), the Coulombic efficiency is increased up to 60% maintaining the OER peak stabilized to the initial curve, which is desirable for a long-term cycling.

In conclusion, in this work we present a novel strategy to obtain porous PEDOT/OIPC scaffolds with electrocatalytic activity toward reversible ORR/OER. Vapor phase polymerization has been used for the preparation of the scaffolds, while the formulation has been optimized with respect to their thermal stability, porosity, morphology, and electrochemical properties. Surprisingly, it has been found that the amount of OIPC in the initial template (in the range studied 7–23 wt %) did not affect the amount of PEDOT estimated through TGA (45 wt % in all the cases). Nonetheless, the morphology of the scaffolds was highly affected by the amount of OIPC, achieving the most porous scaffolds with SC-40 (13 wt % of OIPC) according to BET physisorption and SEM analysis. The porosity was observed to significantly affect the electrochemical response of the scaffolds, affecting not only the ORR activity but also the OER. SC-80 exhibited a more favored ORR than SC-40 with more positive onset potentials while the OER performance was totally different. SC-40 showed a high OER signal, which enabled superior Coulombic efficiencies in basic media, being confirmed in long-term potentiostatic cycling of 200 cycles at 10 mV s^–1^, reaching a Coulombic efficiency of almost 60 wt %. This preparation method of PEDOT/OIPC scaffolds could be used as the baseline for future works to develop electrocatalytic PEDOT porous scaffolds for applications in metal–oxygen batteries.

## Supporting Information Available

Experimental Section; Figure S1: first derivative from the TGA curves of SC-20, SC-40, and SC-80; Table S2: porosity parameters extracted from physisorption experiments; *S*_BET_: specific surface area; *V*_PORE_: pore volume; *D*_PORE_: equivalent pore diameter (calculated as 4 *V*_PORE_/*S*_BET_); Figure S2: differential scanning calorimetry of C_2_mpyrTFSI OIPC, SC-20, SC-40, and SC-80 scaffolds at 10 °C min^–1^; Figure S3: pore size distribution estimated by SEM using ImageJ for 80–100 pores; Figure S4: cyclic voltammograms of PEDOT:PSS, glassy carbon (GC), and platinum (Pt) in N_2_- and O_2_-saturated 0.1 M KOH solution; Figure S5: galvanostatic discharge of SC-40 at 0.05 mA cm^–2^ in 0.1 M KOH electrolyte; electrode mass loading: 47.8 mg cm^–2^; Figure S6: cyclic voltammograms of SC-40, SC-80, and VPP-CNT in O_2_-saturated 0.1 M KOH solution at 10 mV s^–1^ (PDF)

## Abbreviations

EV: electric vehicles
PEDOT: poly(3,4-ethylenedioxythiophene)
ORR: oxygen reduction reaction
OIPC: organic ionic plastic crystal
VPP: vapor phase polymerization
LIB: lithium-ion batteries
OER: oxygen evolution reaction
CNT: carbon nanotubes
CP: conducting polymer
MIEC: mixed ionic and electronic conductor
C_4_mpyr: *N*-butyl-*N*-methylpyrrolidinium
C_2_mpyr: *N*-ethyl-*N*-methylpyrrolidinium
TFSI: bis(trifluoromethanesulfonyl)imide.
