# Supplementary material for: Innovative Strategy for Developing PEDOT Composite Scaffold for Reversible Oxygen Reduction Reaction
Source: J Phys Chem Lett. 2024 Apr 26;15(18):4851–7. doi: 10.1021/acs.jpclett.4c00482 (PMC11089567; doi:10.1021/acs.jpclett.4c00482)
Supplement: Supplementary file 1 — jz4c00482_si_001.pdf [file jz4c00482_si_001.pdf]

Supporting Information

Innovative Strategy for Developing PEDOT Composite Scaffold for Reversible Oxygen Reduction Reaction

Rafael Del Olmo,[1] Antonio Dominguez-Alfaro,[1] Jorge L. Olmedo-Martínez,[1] Oihane Sanz,[2] Cristina Pozo-Gonzalo,[3] Maria Forsyth,[,1,3,4] Nerea Casado*[,1,4]*

[1] POLYMAT, University of the Basque Country UPV/EHU. Joxe Mari Korta Center, Tolosa 72, 20018 Donostia-San Sebastián, Spain.

[2] Department of Applied Chemistry, University of the Basque Country UPV/EHU. 20018, Donostia-San Sebastián, Spain

[3] Institute for Frontier Materials (IFM), Deakin University, Burwood, Victoria 3125, Australia

[4] Ikerbasque, Basque Foundation for Science, E-48011, Bilbao, Spain

E-mail: nerea.casado@ehu.eus, maria.forsyth@deakin.edu.au

1. EXPERIMENTAL SECTION

1.1. Materials

1-Ethyl-1-methylpyrrolidinium bis(trifluoromethylsulfonyl)imide ([C_2mpyr][TFSI]), 99 % was purchased form IoLiTec. 3,4-Ethylenedioxythiophene (EDOT), 99 % was supplied by Fisher Scientific. Iron (III) chloride hexahydrated ($FeCl_3{\cdot}6H_2O$), 99% was acquired from Sigma Aldrich. All the reagents were used as received with no further purification, apart from [C_2mpyr][TFSI], which was dried at 60 ºC under vacuum overnight before use.

1.2. Methods

Thermogravimetric analyses were performed under air (25 mL min^{-1} flow rate) using TGA 8000 Pekin Elmer. The samples were equilibrated at 100 ºC for 20 min and then heated at a rate of 10 ºC min^{-1} in the range of 100-800 ºC. Scanning electron microscope (SEM) measurements were performed on a Hitachi Tabletop Microscope (TM3030 series) at a 15 kV force field, running in a point-by-point scanning mode. The samples were placed on an aluminum holder with double-sided carbon tape and introduced into the SEM chamber. ImageJ was used to measure the pore size distribution in the range of 80-100 pores. The textural properties were characterized by means of N_2 adsorption–desorption at −196 °C in a Micromeritics ASAP2020 apparatus. Prior to the analysis, the materials were degassed at 70 °C during 8 h under vacuum at 10^{-4} mbar. From N_2 adsorption–desorption isotherms, the BET area was calculated from the Brunauer–Emmett–Teller equation. Finally, the pore size distribution (PSD) was calculated using the method proposed by Barrett–Joyner–Halenda (BJH) method.

The electrochemical characterization was carried out using a VMP-3 potentiostat (Biologic Science Instruments). Scaffolds of Ø=5 mm were employed for the ORR glued with 15 µL of PEDOT:PSS (Clevios PH1000) onto a glassy carbon electrode (Ø=4 mm) as working electrode against platinum wire as reference electrode. Cyclic voltammetry was employed in the range of -0.7 to 0.7 V vs Ag/AgCl at different scan rates to observe the electrochemical response of the different materials using 0.1M KOH electrolyte. Platinum wire was used as counter electrode and Ag/AgCl as reference electrode. The liquid electrolyte was saturated with oxygen bubbling O_2 (99.5 %, Air Liquide) for 30 min before running the experiment.

1.3. 3D scaffold synthesis and characterization:

The 3D scaffolds were produced through a multistage process similar to previous reports and as shown in Figure 1.[1,2] Sucrose and OIPC ([C_2mpyr][TFSI]) were sifted through two sieves with mesh sizes of 250 and 100 µm sequentially. Thereafter, sucrose and OIPC grains in the middle

fraction were collected ensuring grain sizes between 250-100 µm. Sieved sucrose (250 mg) and $FeCl_3 \cdot 6H_2O$ (20 mg) oxidant were mixed with the aid of a mortar and pestle in presence of 20 (7wt.%), 40 (13 wt.%) or 80 (23 wt.%) mg of OIPC. Finally, 5 µL of Milli-Q water was added in the blend and subsequently mixed until a homogeneous wet material was obtained. The mixture was poured into a hollow plastic cylinder of (Ø=5 mm) in diameter and gently compacted from both sides to form cylindrical-shape templates of 2-3 mm in length. Then, the templates were hung by a thread inside a Schlenk flask. Afterwards, 0.5 mL of EDOT monomer was introduced at the bottom of the Schlenk flask and subsequently left under vacuum for 5 min. VPP was carried out in a bath of 140 ºC overnight. The temperature of the Schlenk flask at the sucrose-OIPC templates was monitored and did not exceed 55 ºC, which ensures the solid state of the OIPC (T_m = 90 ºC). [32] Once the reaction was completed, the cylinders were immersed overnight into Milli-Q water to dissolve the sucrose and the excess of oxidant, resulting in self-standing and porous architectures with interconnected microchannels. The scaffolds were cleaned with water and isopropanol for five days in a Soxhlet system until complete removal of iron byproducts. The absence of iron was proved by TGA through the complete weight loss.

2. ADDITIONAL MEASUREMENTS

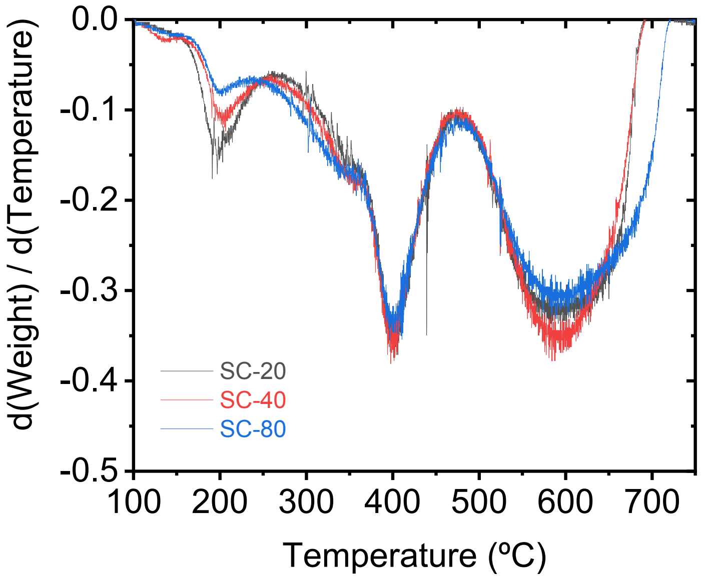

Figure S1. First derivative from the TGA curves of SC-20, SC-40 and SC-80.

Table S1. Porosity parameters extracted from physisorption experiments. S_{BET}: specific surface area; V_{PORE}: pore volume and D_{PORE}: equivalent pore-diameter (calculated as $4V_{PORE}/S_{BET}$).

Scaffold	S_{BET}(m^2 g^{-1})	V_{PORE} (cm^3 g^{-1})	D_{PORE} (nm)
S-20	5.5	0.0311	22.6
S-40	45.2	0.1850	16.4
S-80	10.2	0.0303	11.9

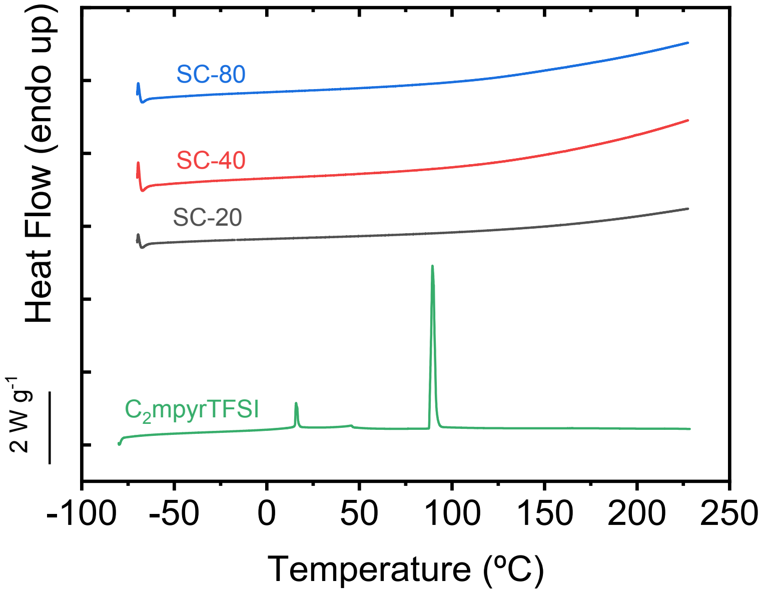

Figure S2. Differential scanning calorimetries of C_2mpyrTFSI OIPC, SC-20, SC-40 and SC-80 scaffolds at 10 ºC min^{-1}.

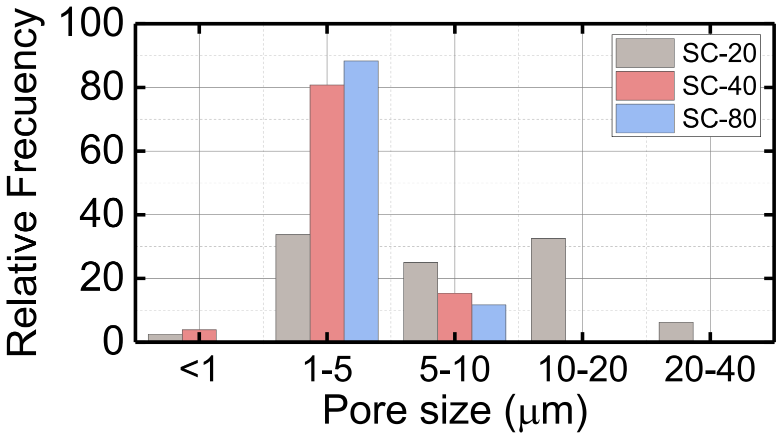

Figure S3. Pore size distribution estimated by SEM using ImageJ for 80-100 pores.

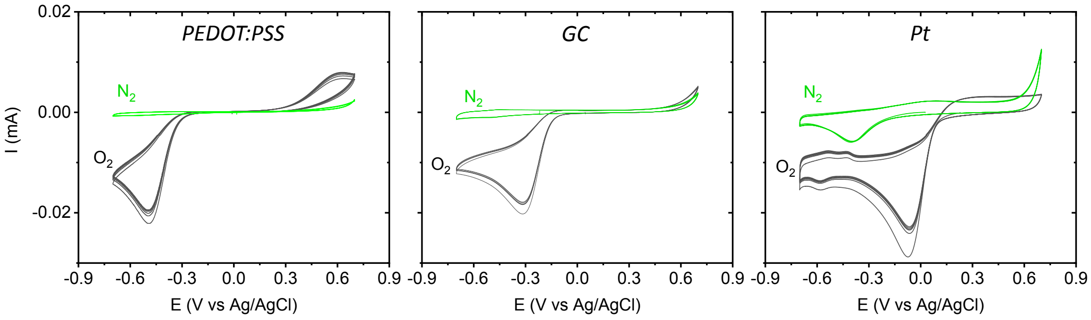

Figure S4. Cyclic voltammograms of PEDOT:PSS, Glassy carbon (GC) and Platinum (Pt) in N_2 and O_2 saturated 0.1 M KOH solution.

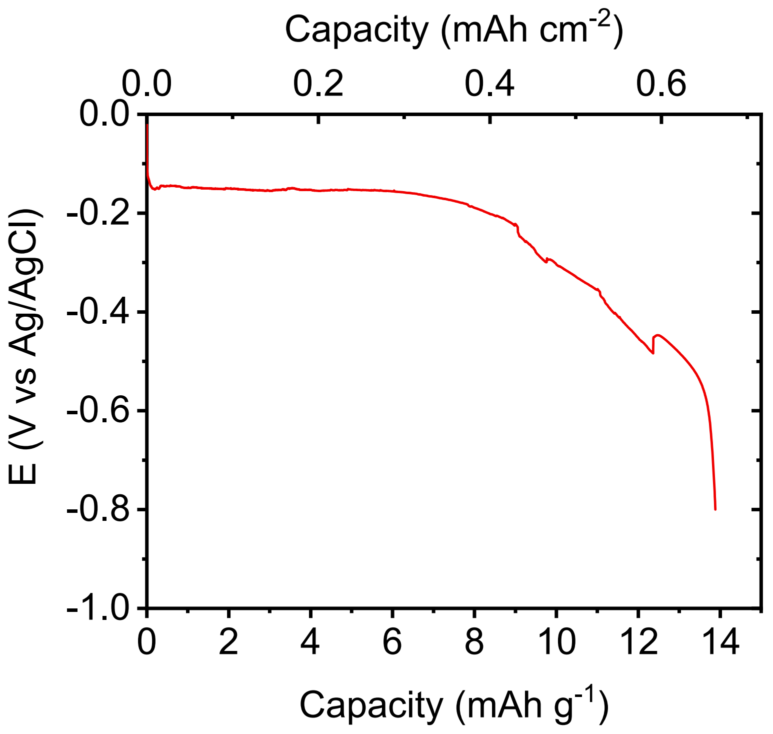

Figure S5. Galvanostatic discharge of SC-40 at 0.05 mA cm^{-2} in 0.1M KOH electrolyte. Electrode mass loading: 47.8 mg cm^{-2}.

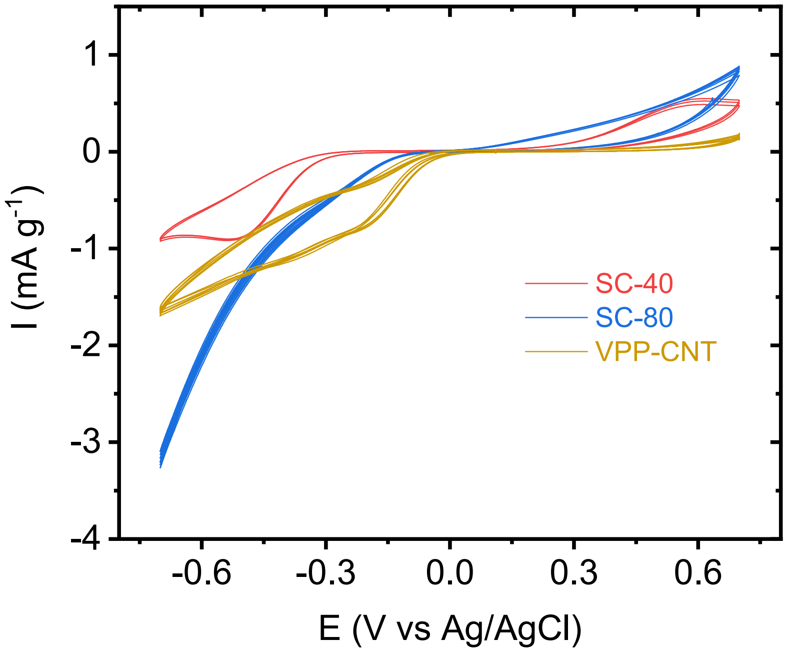

Figure S6. Cyclic voltammograms of SC-40, SC-80 and VPP-CNT in O_2 saturated 0.1 M KOH solution at 10mV s^{-1}.

REFERENCES

(1) Alegret, N.; Dominguez-Alfaro, A.; González-Domínguez, J. M.; Arnaiz, B.; Cossío, U.; Bosi, S.; Vázquez, E.; Ramos-Cabrer, P.; Mecerreyes, D.; Prato, M. Three-Dimensional Conductive Scaffolds as Neural Prostheses Based on Carbon Nanotubes and Polypyrrole. *ACS Appl. Mater. Interfaces* **2018**, *10* (50), 43904–43914.

(2) Dominguez-Alfaro, A.; Alegret, N.; Arnaiz, B.; González-Domínguez, J. M.; Martin-Pacheco, A.; Cossío, U.; Porcarelli, L.; Bosi, S.; Vázquez, E.; Mecerreyes, D.; Prato, M. Tailored Methodology Based on Vapor Phase Polymerization to Manufacture PEDOT/CNT Scaffolds for Tissue Engineering. *ACS Biomater. Sci. Eng.* **2020**, *6* (2), 1269–1278.
